# Supplementary material for: Resolving the full spectrum of human genome variation using Linked-Reads
Source: Genome Res. 2019 Apr;29(4):635–45. doi: 10.1101/gr.234443.118 (PMC6442396; doi:10.1101/gr.234443.118)
Supplement: Supplemental Material [file supp_gr.234443.118_Supplemental_longranger-2.2.zip › longranger-2.2.tar.bz2!/lr2.2-public/lib/stan/cmdstan/stan/doc/api/html/agrad_2fwd_2functions_2log__diff__exp_8hpp_source.html]

Stan: src/stan/agrad/fwd/functions/log\_diff\_exp.hpp Source File


|  |  |
| --- | --- |
|  | Stan  2.5.0  probability, sampling & optimization |


- Main Page
- Related Pages
- Namespaces
- Classes
- Files

- File List
- File Members

All Classes Namespaces Files Functions Variables Typedefs Enumerations Enumerator Friends Macros Pages

- src
- stan
- agrad
- fwd
- functions

log\_diff\_exp.hpp

Go to the documentation of this file.

1 #ifndef STAN\_\_AGRAD\_\_FWD\_\_FUNCTIONS\_\_LOG\_DIFF\_EXP\_HPP

2 #define STAN\_\_AGRAD\_\_FWD\_\_FUNCTIONS\_\_LOG\_DIFF\_EXP\_HPP

3

4 #include <stan/agrad/fwd/fvar.hpp>

5 #include <stan/meta/traits.hpp>

6 #include <stan/math/functions/log\_diff\_exp.hpp>

7 #include <stan/math/constants.hpp>

8

9 namespace stan{

10

11  namespace agrad{

12

13  template <typename T> inline fvar<T>

14  log\_diff\_exp(const fvar<T>& x1, const fvar<T>& x2) {

15  using stan::math::log\_diff\_exp;

16  using stan::math::NOT\_A\_NUMBER;

17  using std::exp;

18  if (x1.val\_ <= x2.val\_)

19  return fvar<T>(NOT\_A\_NUMBER, NOT\_A\_NUMBER);

20  return fvar<T>(log\_diff\_exp(x1.val\_, x2.val\_),

21  x1.d\_ / (1 - exp(x2.val\_ - x1.val\_) ) + x2.d\_ / (1 - exp(x1.val\_ - x2.val\_) ) );

22  }

23

24  template <typename T1, typename T2> inline fvar<T2>

25  log\_diff\_exp(const T1& x1, const fvar<T2>& x2) {

26  using stan::math::log\_diff\_exp;

27  using stan::math::NOT\_A\_NUMBER;

28  using std::exp;

29  if (x1 <= x2.val\_)

30  return fvar<T2>(NOT\_A\_NUMBER, NOT\_A\_NUMBER);

31  return fvar<T2>(log\_diff\_exp(x1, x2.val\_),

32  x2.d\_ / (1 - exp(x1 - x2.val\_)));

33  }

34

35  template <typename T1, typename T2> inline fvar<T1>

36  log\_diff\_exp(const fvar<T1>& x1, const T2& x2) {

37  using stan::math::log\_diff\_exp;

38  using stan::math::NOT\_A\_NUMBER;

39  using std::exp;

40  if (x1.val\_ <= x2)

41  return fvar<T1>(NOT\_A\_NUMBER, NOT\_A\_NUMBER);

42  return fvar<T1>(log\_diff\_exp(x1.val\_, x2),

43  x1.d\_ / (1 - exp(x2 - x1.val\_)));

44  }

45  }

46 }

47 #endif

stan::math::log\_diff\_exp

boost::math::tools::promote\_args< T1, T2 >::type log\_diff\_exp(const T1 x, const T2 y)

The natural logarithm of the difference of the natural exponentiation of x1 and the natural exponenti...

**Definition:** log\_diff\_exp.hpp:49

stan::agrad::log\_diff\_exp

fvar< T > log\_diff\_exp(const fvar< T > &x1, const fvar< T > &x2)

**Definition:** log\_diff\_exp.hpp:14

stan::math::NOT\_A\_NUMBER

const double NOT\_A\_NUMBER

(Quiet) not-a-number value.

**Definition:** constants.hpp:53

stan::agrad::fvar

**Definition:** fvar.hpp:13

traits.hpp

log\_diff\_exp.hpp

fvar.hpp

stan::agrad::fvar::d\_

T d\_

**Definition:** fvar.hpp:16

constants.hpp

stan::agrad::exp

fvar< T > exp(const fvar< T > &x)

**Definition:** exp.hpp:16

stan::agrad::fvar::val\_

T val\_

**Definition:** fvar.hpp:15


---

[ Stan Home Page ]

*© 2011–2014,
Stan Development Team.*
